# Midkine and NANOG Have Similar Immunohistochemical Expression Patterns and Contribute Equally to an Adverse Prognosis of Oral Squamous Cell Carcinoma

**DOI:** 10.3390/ijms18112339

**Published:** 2017-11-06

**Authors:** Hyun-Min Kim, Young-Hoon Kang, June-Ho Byun, Si-Jung Jang, Gyu-Jin Rho, Jong-Sil Lee, Bong-Wook Park

**Affiliations:** 1 Department of Dentistry, School of Medicine and Institute of Health Science, Gyeongsang National University, Jinju 52727, Korea; acerhm77@gmail.com (H.-M.K.); younghoon300@hanmail.net (Y.-H.K.); surbyun@gnu.ac.kr (J.-H.B.); 2 Department of Oral and Maxillofacial Surgery, Changwon Gyeongsang National University Hospital, Changwon 51472, Korea; 3 OBS/Theriogenology and Biotechnology, College of Veterinary Medicine, Gyeongsang National University, Jinju 52828, Korea; sjjang@gnu.ac.kr (S.-J.J.); jinrho@gnu.ac.kr (G.-J.R.); 4 Department of Pathology, School of Medicine Gyeongsang National University, Jinju 52727, Korea

**Keywords:** oral squamous cell carcinoma, immunohistochemistry, MIDKINE, NANOG

## Abstract

To increase the overall survival rate and obtain a better prognosis for oral squamous cell carcinoma (OSCC) patients, the detection of more effective and reliable tumor prognostic markers is needed. This study is focused on the analysis of correlation between the clinicopathological features of OSCCs and the immunohistochemical (IHC) expression patterns of MIDKINE (MK) and NANOG. Sixty-two primary OSCC patients were selected and their pretreatment biopsy specimens were immunohistochemically analyzed for the MK and NANOG proteins. The IHC expression patterns, clinicopathological features, and overall survival rates were assessed to identify any correlations. MK and NANOG showed significantly similar IHC expression patterns: both demonstrated enhanced expression in histologically high-grade and clinically late-stage OSCCs. Weak or negative expression of MK and NANOG was correlated with negative neck node metastasis. Clinicopathologically, late tumor stage, neck node metastasis, high-grade tumor, and palliative treatment groups showed significantly lower overall survival rates. The enhanced expression of MK and NANOG was associated with lower overall survival rates. In particular, enhanced co-detection of MK and NANOG showed significant correlation with poor prognosis. In conclusion, enhanced IHC expression patterns of MK and NANOG in OSCC patients was significantly associated with lower overall survival rates and unfavorable clinicopathological features. These results demonstrate that analysis of IHC expression patterns of MK and NANOG in pretreatment biopsy specimens during the work-up period can provide a more definitive prognosis prediction for each OSCC patient that can help clinicians to develop a more precise individual treatment modality.

## 1. Introduction

Oral squamous cell carcinoma (OSCC) is the most common type of oral cancer, and comprises at least 90% of all oral malignancies [1,2]. Despite intensive research into OSCC, the overall 5-year survival rate is less than 50%, and has remained largely unchanged over the past 40 years [3,4]. Because the traditional TNM (tumor, node, metastases) classification system and histological grade are limited in their ability to accurately predict the tumor’s aggressiveness and prognosis, various studies have been performed for the detection of more effective prognostic markers in OSCC patients [1,5,6,7]. In previous reports, cancer stem cells (CSCs) were shown to have multiple functions in carcinogenesis, differentiation and in contributing to the heterogeneity of tumor cells [4,5,6,7]. Moreover, the expression levels of CSCs showed a direct relationship with clinicopathological status and prognosis of various tumors [4,7,8]. Interestingly, embryonic stem cell (ESC) markers such as NANOG, OCT4, SOX2, and STAT3 showed high expression and played important roles in CSCs of OSCC [4,8,9]. In particular, OCT4, NANOG, and SOX2 are considered key regulators for the self-renewal and maintenance of stemness of CSCs as well as ESCs [4]. Recently, reports have shown that high IHC expression of NANOG and OCT4 is correlated with histopathologically high-grade tumors and clinically poor prognoses [8,9,10,11,12]. Similarly, our previous study showed that increased expression of NANOG and mutant p53 in pretreatment OSCC specimens was significantly associated with clinically late-stage and histologically high-grade tumors, positive neck node metastasis, and poor overall survival rates [7]. To develop a personalized optimal treatment modality for each OSCC patient, a complete analysis of each tumor’s properties, including its aggressiveness and predicted course, during the initial diagnostic work-up period is an important process that may result in a better prognosis after cancer treatment. Pretreatment analysis of a cancerous biopsy specimen can provide a large amount of information regarding tumor characteristics.

Interestingly, recent studies demonstrate that one of the growth factors, MIDKINE (MK), has a close relationship with cancer development at various sites [2,13]. Especially, MK was highly expressed in OSCC patients in the serum level as well as cancer tissue [13,14,15]. MK is the product of a retinoic acid-response gene and belongs to the family of heparin-binding growth and differentiation factors [2,16,17]. Various roles of MK have been reported, including promotion of cell proliferation, migration, and survival [17]. In particular, recent reports have demonstrated that aberrant expression of MK is strongly associated with carcinogenesis in various cancers, and suggest that it could be used as a novel biomarker for cancer [13,14,18,19].

The present study focuses on the analysis of correlation between clinicopathological features of OSCC patients and immunohistochemical (IHC) expression patterns of MIDKINE (MK) and NANOG. In this study, we demonstrated that the IHC expression patterns of MK and NANOG are highly similar in pretreatment biopsy specimens of OSCCs, and that both markers were strongly expressed in histopathologically high-grade tumors and clinically late-stage OSCCs. In addition, the expression patterns of MK and NANOG were correlated with the overall survival rate of OSCC patients, indicating that the IHC expression pattern of MK and NANOG in pretreatment biopsy specimens can be used as a tumor prognosis marker in these patients.

## 2. Results

### 2.1. Selected Patient Information

A total of 62 patients with OSCC consisting of 40 males and 22 females were included in this study. Patient age ranged between 17–91 years (mean: 65.6 ± 13.8 years). The site of OSCC in the patients included the gingiva (28 cases), palate (11 cases), tongue (10 cases), floor of the mouth (7 cases), and cheek (6 cases) (Table 1). In the present study, patients were divided into three groups according to the main treatment modality: surgery-only group (Surg), surgery combined with adjuvant radiotherapy or concurrent chemoradiotherapy group (Surg + RT), and palliative radiotherapy or no treatment group (PaRT). Most patients in the PaRT group underwent palliative radiotherapy only or received hospice treatment because they refused surgery or because their tumors were inoperable. The number of patients in each group is given as follows: 18 patients in the Surg group, 22 patients in the Surg + RT group, and 22 patients in the PaRT group. The mean follow-up period in all patients was 33.5 months. Twenty-nine patients (22, 3, and 4 in the PaRT, Surg + RT, and Surg group, respectively) died after an average of 19 months, and 33 patients (19 and 14 in the Surg + RT and Surg group, respectively) survived with an average follow-up of 46.2 months. In all cases of surgery, tumor margins for resection were set as at least 2 cm, and the resection field was confirmed to be tumor-free using frozen biopsy sections.

### 2.2. Expression Patterns of NANOG and MK in OSCC Pretreatment Biopsy Specimens

**Figure 1:**
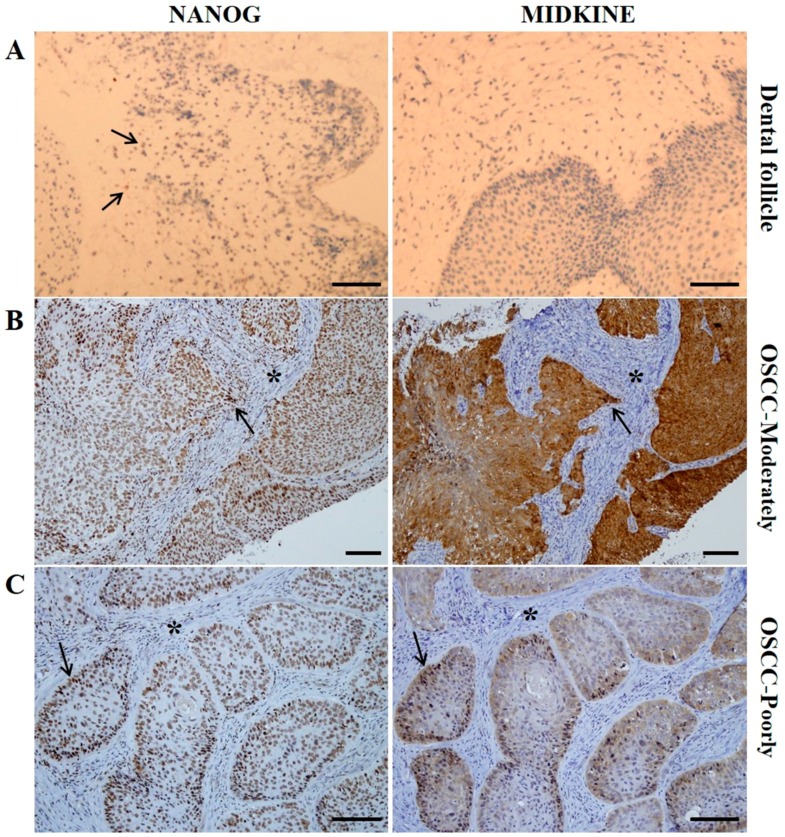
Results of IHC staining of MIDKINE (MK) and NANOG in control and OSCC specimens. (**A**) In dental follicle tissue from extracted wisdom tooth (control), MK is negatively expressed, whereas some cells show weak expression of NANOG proteins (**arrows**), which might be related to undifferentiated mesenchymal stem cells in dental follicle tissue; (**B**,**C**) in serial sections of high-grade (moderately- and poorly-differentiated) OSCC specimens, MK and NANOG proteins showed highly similar expression patterns, were negatively expressed in interstitial tissue (*) and strongly expressed in cancer cells. NANOG is predominantly expressed in the nuclei of cancer cells but is usually detected in the cytoplasm, indicating high stemness of these cancer cells. MK is mainly expressed in the cytoplasm of cells but it is also highly detected in nuclei, which might be related to the higher proliferation rate and increased survivability of cancer cells. Interestingly, a large proportion of OSCC cells in high-grade tumors showed strong co-detection of MK and NANOG (arrows). Scale bar = 50 µm.

**Figure 2:**
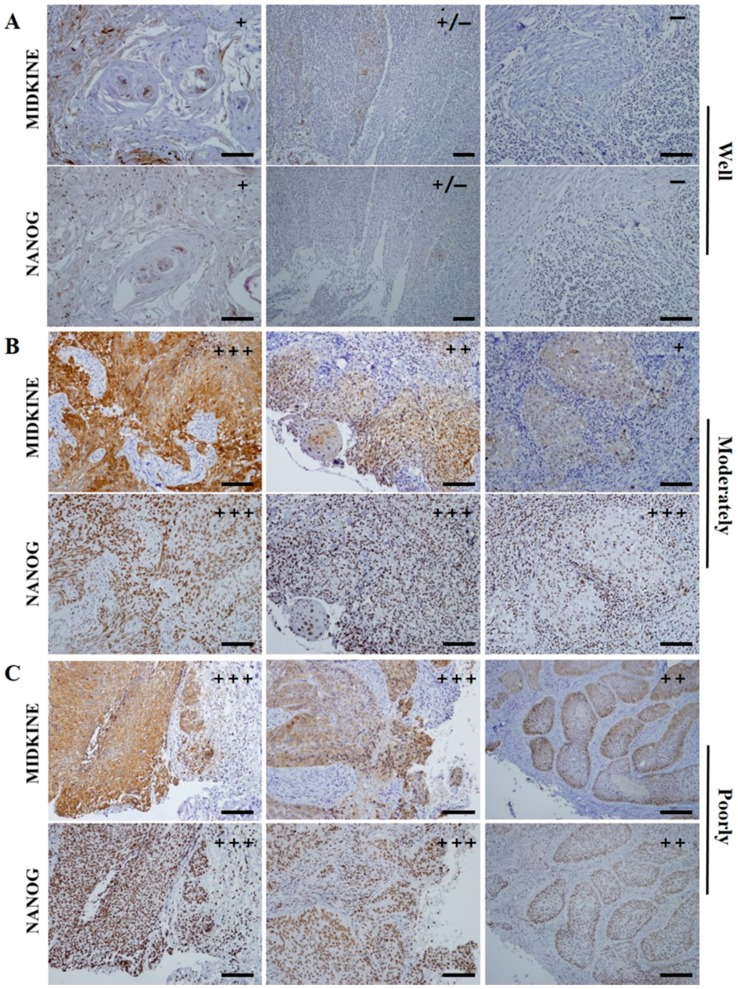
Representative serial section images of IHC staining of MK and NANOG in the pretreatment OSCC biopsy specimens as their histopathological grades. (**A**) In well differentiated OSCCs, weak or negative expression of MK and NANOG is usually detected; (**B**,**C**) high-grade tumors, moderately- or poorly-differentiated OSCCs, dominantly revealed the enhanced (strong and moderate) expressions of MK and NANOG proteins. The expression patterns these two marker proteins, including immunostaining intensity and positive cell ratio, revealed highly similar features in OSCC specimens. In particular, enhanced co-localization of MK and NANOG proteins was usually detected in the cancer cells of high-grade tumors. Scale bar = 100 µm.

**Figure 3:**
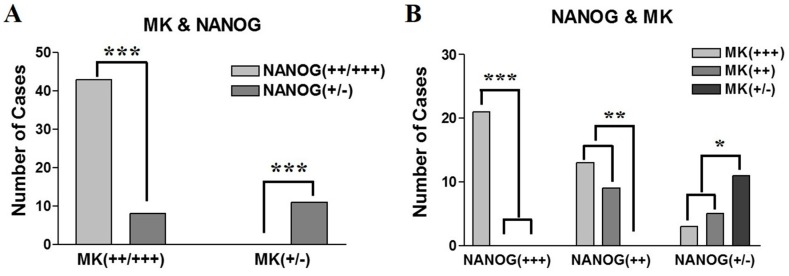
Graphs showing the correlation between IHC expression patterns of MK and NANOG in pretreatment OSCC specimens. (**A**) The enhanced expression (strong or moderate) of MK is significantly correlated with high expression of NANOG (*p* < 0.001), whereas weak or negative expression of MK is positively associated with weak expression of NANOG (*p* < 0.001); (**B**) similarly, strong or moderate expression of NANOG protein dominantly possess the enhanced MK protein (*p* < 0.01), and weak expressions of NANOG and MK have a positive relationship (*p* < 0.05). Data represent the number of cases with positive expression of each protein, and asterisks indicate statistical significances (* *p* < 0.05; ** *p* < 0.01; *** *p* < 0.001).

In control specimens—dental follicle tissues from extracted wisdom teeth—there was no expression of MK; however, a small population of dental follicle cells showed a weak positive expression of NANOG (Figure 1A). This is coincident with results from our previous study, in which we showed that dental follicle tissue has undifferentiated mesenchymal stem cells which can express the pluripotent markers OCT4, SOX2, and NANOG [20]. In pretreatment biopsy specimens of OSCC, NANOG was mainly detected in the nuclei of OSCC cells, but some NANOG expression was detected in the cytoplasm of cancer cells (Figure 1B,C). In contrast, MK was predominantly localized in the cytoplasm of cancer cells, but it was usually found in both nuclei and cytoplasm in sites of enhanced expression (Figure 1B,C). The number of positive cells and the expression intensities were usually greater in high-grade OSCCs (moderately- or poorly-differentiated) than in low grade tumors (well-differentiated). Enhanced expression of MK and NANOG was mostly detected in high-grade tumors, whereas they were usually weakly or negatively expressed in low grade OSCCs (Figure 2). In serial sections of high-grade OSCC specimens, MK and NANOG proteins showed highly similar expression patterns, in which they were negatively expressed in interstitial tissue and strongly expressed in cancer tissues and cells (Figure 1B,C and Figure 2B,C). In particular, enhanced co-localization of MK and NANOG proteins was usually detected in the cancer cells of high-grade tumors (arrows in Figure 1). Of the 62 OSCC specimens, the expression of NANOG was strong in 21 specimens, moderate in 22 specimens, and weak or negative in 19 specimens. Similarly, strong, moderate, and weak or negative expression of MK was observed in 37, 14, and 11 OSCC specimens, respectively (Table 1). Direct relationships between the expression intensities of NANOG and MK were observed (Figure 3). Most tumors with enhanced (strong or moderate) expression of MK also exhibited enhanced NANOG expression (*p* < 0.001). In addition, a strong relationship between weak or negative expressions of MK and NANOG was observed (*p* < 0.05) (Figure 3A,B).

### 2.3. Association Between Clinicopathological Features and the Expression Patterns of NANOG and MK

**Figure 4:**
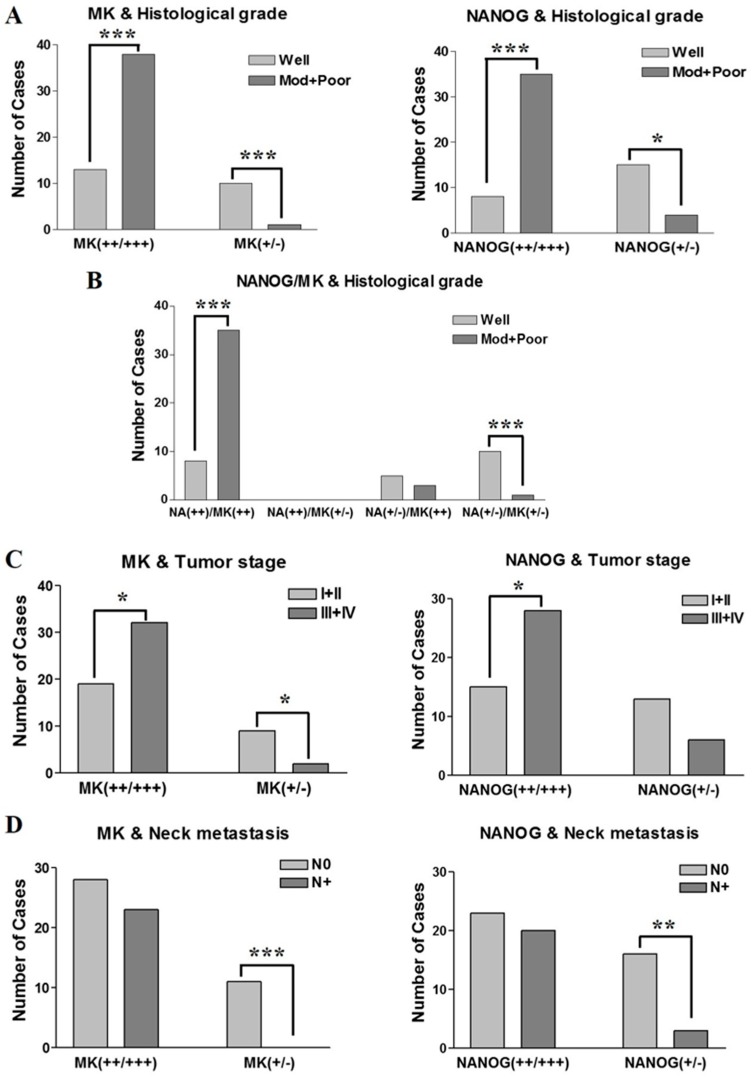
Correlation between IHC expression patterns of MK and NANOG and clinicopathological features of OSCCs. (**A**) The enhanced expression of MK and NANOG proteins showed strong correlation with high-grade OSCC (*p* < 0.001), whereas weak or negative expression of the two markers is significantly correlated with a well-differentiated tumor (MK: *p* < 0.001; NANOG: *p* < 0.05); (**B**) in particular, co-enhanced expression of MK and NANOG is significantly correlated with moderately- or poorly-differentiated high-grade OSCC (*p* < 0.001). Conversely, weak or negative co-expression of MK and NANOG is predominantly detected in well-differentiated tumors (*p* < 0.001); (**C**) graphs show the enhanced expression of MK and NANOG is directly associated with late-stage tumors (stage III or IV) (*p* < 0.05), whereas weak or negative expression of two markers is associated with early-stage tumors (stage I or II) (*p* < 0.05); (**D**) graphs show the weak or negative expression of MK or NANOG protein associated with negative neck node metastasis (MK: *p* < 0.001; NANOG: *p* < 0.01), although the enhanced expression of two markers has no association with positive neck node metastasis (*p* > 0.05). Data represent the number of cases with positive expression of each protein, and asterisks indicate statistical significance (* *p* < 0.05; ** *p* < 0.01; *** *p* < 0.001).

In the present study, NANOG and MK have similar IHC expression patterns in pretreatment biopsy specimens of OSCC (Figure 1 and Figure 2), which show the same association with clinicopathological features. Strong expression of NANOG or MK was significantly correlated with histologically high-grade OSCCs (*p* < 0.001), whereas weak or negative expression of NANOG (*p* < 0.05) or MK (*p* < 0.001) predominantly occurred in well-differentiated carcinomas (Figure 4A). Interestingly, the IHC co-expression pattern of NANOG and MK represented a stronger association with tumor histological grade, and strong co-detection of the two marker proteins was observed in high-grade OSCC. However, weak or negative co-expression was significantly associated with well-differentiated tumors (*p* < 0.001) (Figure 4B).

In the assessment of the relationship between protein expression and tumor stage, strong and moderate expression of NANOG or MK displayed direct associations with late-stage tumors (stage III and IV; *p* = 0.047 and 0.048 for NANOG and MK, respectively). Moreover, weak expression of MK or NANOG in the present study was frequently detected in early-stage tumors (stage I and II), but a statistical difference was only found for weak MK expression (*p* = 0.035 and *p* > 0.05 for MK and NANOG, respectively) (Figure 4C). In the analysis of neck node metastasis, the enhanced expression of NANOG or MK had no association with positive neck node metastasis, but weak or negative expression of NANOG or MK was significantly associated with negative neck node metastasis in OSCCs (*p* = 0.003 and *p* < 0.001 for NANOG and MK, respectively) (Figure 4D).

### 2.4. Survival Analysis

**Figure 5:**
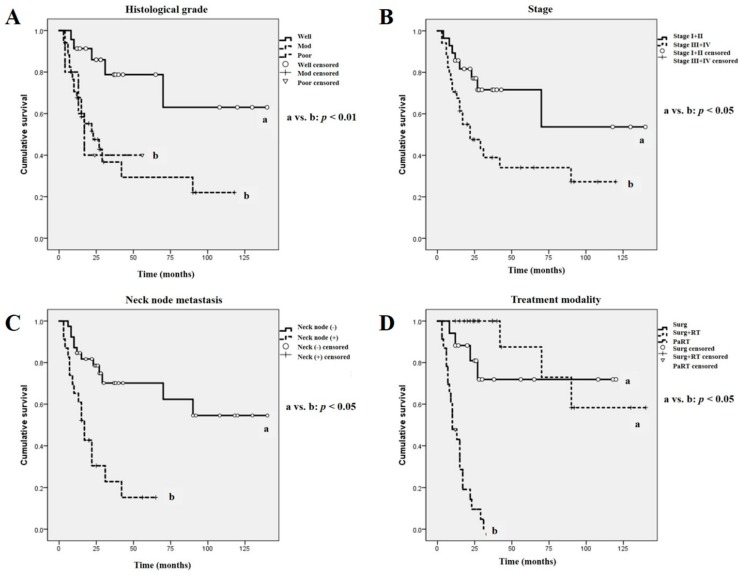
The overall survival rate of OSCCs according to clinicopathological features and treatment modality. (**A**–**D**) OSCC patients with well-differentiated tumors (*p* < 0.01), early-stage tumors (*p* < 0.05), negative neck node metastasis (*p* < 0.05), and surgical treatment (Surg and Surg + RT) groups (*p* < 0.05) showed significant higher overall survival rates than those in the corresponding opposite groups. Different letters denote statistically significant differences between groups (*p* < 0.05 or *p* < 0.01).

The analysis of overall patient survival rates in terms of clinicopathological tumor features revealed some significant results, similar to our previous report [7] (Figure 5). Patients with histopathologically high-grade (moderately- or poorly-differentiated) OSCC had a lower overall survival rate than those with well-differentiated low grade tumors (*p* < 0.01, Figure 5A). In addition, patients with late-stage OSCC (stage III and IV) had a statistically significant lower survival rate than those with early-stage tumors (Stage I and II) (*p* < 0.05, Figure 5B). Patients with OSCC and positive neck node metastasis displayed a significantly poorer survival rate than those with negative neck node metastasis (*p* < 0.05, Figure 5C). Moreover, in terms of treatment modality, the PaRT treatment group exhibited a significantly poorer survival rate than the Surg and Surg + RT groups (*p* < 0.05, Figure 5D).

**Figure 6:**
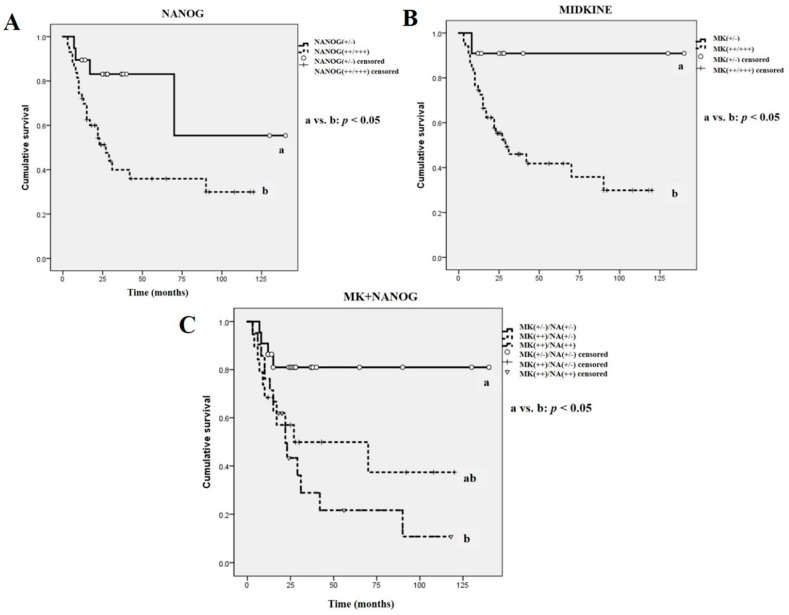
The overall survival rate of OSCC patients according to IHC expression patterns of MK and NANOG. (**A**,**B**) OSCC patients with enhanced expression (strong or moderate) of MK or NANOG show significantly lower overall survival rates than those with weak or negative expression of the two marker proteins (*p* < 0.05); (**C**) In particular, enhanced co-expression of MK and NANOG [MK(++)/NANOG(++)] in the pretreatment OSCC specimens showed significant correlation with lower overall survival rate than those with weak or negative co-expression of MK and NANOG [MK(+/−)/NANOG(+/−)] (*p* < 0.05). Different letters denote statistically significant differences between groups (*p* < 0.05).

Moreover, survival analysis in terms of the immunostaining patterns of NANOG and MK revealed statistically significant differences (Figure 6). The expression intensity of NANOG was directly associated with the overall survival rate; patients with enhanced NANOG-expressing tumors [NANOG(+++/++)] had poorer survival rates than those with weak or negative NANOG-expressing tumors [NANOG(+/−)] (*p* < 0.05, Figure 6A). Similarly, a positive association between MK expression and the overall survival rate was observed; strong or moderate expression of MK [MK(+++/++)] had lower survival rates than those with weak or negative MK-expressing OSCCs [MK(+/−)] (*p* < 0.05, Figure 6B). Interestingly, patients with enhanced co-expression of NANOG and MK (moderate or strong expression) [NANOG(++)/MK(++)] had significantly lower survival rates than patients with weak or negative co-expression of NANOG and MK [NANOG(+/−)/MK(+/−)] (*p* < 0.05, Figure 6C). Significantly, among the 43 patients with NANOG(++)/MK(++) tumors, there were 25 patient deaths during the follow-up period, whereas in the 11 patients with NANOG(+/−)/MK(+/−) tumors there was only one death, which was that of the oldest patient (male, 91 years) who had received only palliative care and died 8 months after diagnosis. In addition, univariate analysis (Kaplan–Meier survival analysis) and revealed that neck node metastasis, histologically high-grade tumor, and late tumor stage were directly associated with a significantly lower survival rate. Moreover, enhanced expression of NANOG and MK, especially co-detection of these two proteins, in the OSCC pretreatment biopsy specimens was associated with a poorer prognosis (Table 2). Similarly, the multivariate Cox proportional hazards regression model illustrated that neck node metastasis, tumor histological grade, and IHC expression patterns of NANOG and MK, including co-enhanced expression of two markers, were directly related to the overall survival rate of patients with OSCC (Table 3).

## 3. Discussion

In the present study, MK has a highly similar IHC expression pattern to NANOG in pretreatment OSCC specimens. The increased expression of MK and NANOG was associated with histologically high-grade tumors (moderately- and poorly-differentiated OSCC) and clinically adverse prognosis (late tumor stage and lower overall survival rate), compared to tumors with weak or negative expression of MK and NANOG. Moreover, weak or negative expression of these markers was associated with negative neck node metastasis. In particular, the co-expression pattern of MK and NANOG showed strong correlation with histopathological grade and overall survival rate of OSCC.

Previous studies have reported that overexpression of MK in many kinds of cancer specimen, including esophageal, pancreatic, colorectal, lung, and breast carcinomas [13,14,21,22]. Study of MK expression in OSCC specimens began relatively recently. Ruan et al. (2007) first reported the correlation between the IHC expression pattern of MK in OSCC specimens with clinicopathological features and tumor angiogenesis, and concluded that high MK expression is positively correlated with late tumor stage, larger tumor size, and poor prognosis as well as higher tumor angiogenesis [15]. Similarly, other researchers demonstrated that enhanced MK expression showed adverse prognosis with increased neck node metastasis and late clinical tumor stage [2,19,23]. Interestingly, serum MK levels were also useful for predicting cancer prognostic markers in OSCC patients, and it was concluded that increased serum MK concentration in early-stage OSCCs were strongly associated with poor survival [14]. In addition, MK has been shown to mediate intercellular cytoprotective signals that contribute to the development of resistance to chemotherapy [2,24,25]. Conversely, other reports have demonstrated that downregulation of MK induced cisplatin resistance in OSCC cell lines, which might be related to the inhibition of cell growth and the decreasing cellular accumulation of cisplatin when MK was suppressed in an OSCC cell line [23]. Even though the exact mechanism was not understood, these ambiguous characteristics of MK regarding drug resistance could be related to its concentration and/or the properties of the target drug. These results also indicate that MK may play a pivotal role in drug resistance in OSCC through modulation of its cytoprotective properties, cell proliferation, or drug accumulation [23].

Until now, the exact mechanism through which MK is regulated in cancer cells has not been revealed. Some genes and proteins, including the nuclear factor-κB (NF-κB), hypoxia-inducible factor-1a (HIF-1a), Deleted in azoospermia-like gene (DAZL), and thyroid transcription factor-1 (TTF-1), have been identified as regulating factors of MK expression [17,23,26]. Recently, it was revealed that the tumor suppressor gene p53 is one of the main regulating factors of MK [2]. OSCC samples with higher expression of mutant p53 and lower levels of wild-type p53 showed increased expression of MK [2]. In addition, the transcriptional activity of the MK promoter was shown to be regulated by a p53-dependent pathway [27]. The other important role of MK in tumor cell growth is its role in promoting angiogenesis. Tumors expressing enhanced MK were more likely to have higher microvessel density (MVD), indicating that MK might have an important function in tumor angiogenesis [15,28]. However, there have been conflicting results for the correlation between MK and vascular endothelial growth factor (VEGF) in cancer cells: some researchers reported MK expression was significantly correlated with VEGF expression in various cancer cells [21,22], whereas others observed that MK decreased VEGF-A-induced neovascularization and vascular permeability [29]. In addition, MK increased MVD in OSCC specimens despite low levels of VEGF, indicating that MK could promote angiogenesis via a VEGF-independent pathway [15]. Although further investigation is needed to determine the precise relationship between MK, angiogenesis and VEGF, it is apparent that MK is able to increase vessel density and angiogenesis in cancer tissue, which contributes to rapid growth and proliferation of tumor cells.

NANOG, an early transcription factor, is a pluripotent marker of stem cells that is involved in functionally blocking differentiation and thus maintains the self-renewal ability and pluripotency of ESCs and mesenchymal stem cells [4,9]. Many recent studies have reported that high expression of NANOG in OSCC specimens is directly associated with histologically poor differentiation status, clinically late-stage tumors, and frequently with neck node metastasis, resulting in poor overall survival rates [4,7,9,10,11,12]. However, to the best of our knowledge, the correlation between NANOG and MK expression in cancer specimens has not been studied. In the present study, we observed that IHC expression of NANOG and MK revealed highly similar patterns in pretreatment OSCC biopsy specimens, and that enhanced expression was predominantly detected in moderately- and poorly-differentiated tumors, whereas they were usually weakly- or negatively-expressed in well-differentiated OSCC. In immunohistochemistry, NANOG is usually detected in the nuclei, but MK is mainly localized in the cytoplasm of pluripotent stem cells or cancer cells [2,7,15]. However, NANOG has occasionally been detected in the cytoplasm of highly primitive cells or invasive cancer cells [7,30,31]. These findings may be related to the high pluripotency of undifferentiated stem cells or cancer cells [30,31]. Similarly, MK has also been shown to be highly expressed in the nuclei of high-grade cancer cells; elevated MK expression with nuclear targeting is usually associated with the promotion of cell survival [2,28,32]. These aberrant expression patterns of MK and NANOG may be related to the high carcinogenicity with an increased proliferation rate of OSCC cells. In the present study, cytoplasmic expression of NANOG and nuclear detection of MK were usually observed in high-grade, moderately- or poorly-differentiated, OSCC specimens with enhanced expression of these two marker proteins. In addition, elevated and aberrant expression patterns of NANOG and MK proteins have been associated with late-stage tumors and poor overall survival rates. In particular, strong co-detection of NANOG and MK showed a high association with adverse prognosis in patients with OSCC. Interestingly, in the present study, MK was not detected but weak NANOG staining was observed in a small population of dental follicle cells from extracted wisdom teeth (control specimen). These results are in agreement with those from previous reports, in which MK was not detected in normal oral mucosa [15]. However, dental follicles from extracted wisdom teeth possess undifferentiated mesenchymal stem cells that express the pluripotent markers, OCT4, SOX2 and NANOG [20].

Although further studies, including in vitro OSCC cell line experiments, are needed to determine the precise relationship between MK and NANOG in OSCC, it is clear that these two marker proteins have highly similar IHC expression patterns, and that enhanced detection of MK and NANOG is strongly correlated with an adverse prognosis. Similarly, our previous study showed that positive expression of NANOG and mutant p53 by immunohistochemistry was directly associated with unfavorable clinicopathological features and poor prognosis of OSCC [7]. Taken together, these results indicate that analysis of the IHC expression patterns of NANOG, mutant p53, and MK in pretreatment OSCC specimens can provide a more definitive prognosis prediction, which can help clinicians to develop a more precise individual treatment modality.

## 4. Materials and Methods

### 4.1. Patients

Patient selection criteria were similar to those in our previous report [7]. Briefly, a total of 62 patients (40 males and 22 females) were selected for the present study, all of whom were diagnosed with primary OSCC between 2006 and 2016 in the Department of Oral and Maxillofacial Surgery of Gyeongsang National University Hospital (GNUH) and Changwon Gyeongsang National University Hospital. Diagnosis was made on the basis of histopathological analysis of biopsy specimens collected prior to any application of cancer treatment. The inclusion criteria were: patients who gave informed consent to participate in the study, those who had completed at least 3 months of follow-up after undergoing a pretreatment biopsy, and those who had sufficient tissue volume in the pretreatment biopsy specimen to undergo IHC staining. Patients with metastatic OSCC and/or recurrent tumor, less than 3 months follow-up, and/or insufficient volume of biopsy specimen were excluded from the present study.

The patients’ medical records were retrospectively analyzed. All patients provided informed consent for the use of their tissue specimens, and the study was approved by the Ethics Committee for Clinical Research at GNUH (GNUH IRB-2012-09-004-002, 12-10-2012). Immunohistochemistry was performed on the pretreatment biopsy specimens of the 62 patients. Under local anesthesia with 2% Lidocaine (1:100,000 epinephrine) in the tumor site, the incisional biopsy was obtained from the periphery to the center of tumor with a minimum length of 5 mm and a minimum width and depth of 2 mm. The biopsy specimens were immediately fixed with 10% neutral buffered formalin and were sent to the pathology department to procedure IHC analysis. The duration of follow-up for the 62 patients ranged from 3 to 140 months, with a mean of 33.5 months. The patients were clinically evaluated according to the TNM classification system developed by the American Joint Committee on Cancer (7th edition, 2010) for tumor stage and neck node metastasis, and overall survival rates were also determined. Biopsy specimens were histopathologically graded, and tumors were categorized as well-, moderately-, or poorly-differentiated, according to the WHO guidelines for tumor classification [33]. Patients were divided into three groups according to main treatment modality: a surgery only group (Surg), a surgery combined with adjuvant radiotherapy or concurrent chemoradiotherapy group (Surg + RT), and a palliative radiotherapy or no treatment group (PaRT). Most patients in the PaRT group underwent palliative radiotherapy only or received hospice treatment because they refused to undergo surgery or their tumors were inoperable.

### 4.2. Immunohistochemical Analysis

IHC analysis of biopsy specimens was performed using the same method as in our previous study [7]. Briefly, pretreatment biopsy specimens of OSCC were fixed in 10% neutral buffered formalin for 24 h, embedded in a paraffin block, sliced into 4-µm sections and mounted on SuperfrostPlus microscope slides (Fisher Scientific, Rochester, NY, USA). The sections were maintained at room temperature for 12 h. After hydration, immunohistochemistry for NANOG and mutant MK was performed using an automated immunostainer (BenchMark XT, Ventana Medical Systems Inc., Tucson, AZ, USA), and visualization was conducted using the Ultraview DAB kit (Ventana Medical Systems Inc.) according to the manufacturer’s protocol. For immunostaining of the proteins, 1:250 diluted rabbit monoclonal anti-Nanog (ab109250, Abcam™, Cambridge, UK) and 1:200 diluted rabbit monoclonal anti-Midkine (ab52637, Abcam™) were used as primary antibodies.

Briefly, sections were deparaffinized using EZ Prep solution; then CC1 standard (pH 8.4 buffer containing Tris/Borate/EDTA) was applied for antigen retrieval for 60 min at 100 °C. The slides were incubated at 37 °C for 4 min with a DAB inhibitor (3% H_2_O_2_) to block endogenous peroxidase activity. Slides were then incubated with the primary antibody at 37 °C for 32 min, followed by incubation with the secondary antibody (Universal HRP Multimer) for 8 min at 37 °C. Slides were treated with DAB + H_2_O_2_ substrate for 8 min, followed by hematoxylin II and bluing reagent at 37 °C for nuclear counterstaining. For negative controls, only incubation with the secondary antibody was permitted, omitting any incubation with a primary antibody, in sections from the same specimens and under the same conditions. In addition, human embryonic urinary bladder tissue was used as a positive control for both NANOG and MK according to the antibody manufacturer’s recommendations.

Tissue slices were semi-quantitatively analyzed for antibody binding in cellular components by two pathologists who were blinded to the study information to avoid bias for the present study. Based on a previously reported method [7,11], positive immunostaining of NANOG and MK was scored with a combination of intensity (where 0, 1, 2 and 3 represent negative, weak, moderate and strong staining, respectively) and the percentage of positively stained tumor cells in high-power fields (0, negative; 1, <25%; 2, 25–50%; 3, 51–75%; and 4, >75%). The sum of the staining intensity and percentage of positive tumor cell scores was graded as follows: +++ (strong, 6–7); ++ (moderate, 4–5); + (weak, 2–3); and − (negative, 0–1). At least three different fields under high magnification (×400) per slide were analyzed for immunostaining intensity. A summary of the IHC staining results can be found in Table 1.

### 4.3. Statistical Analysis and Overall Survival Analysis

The affiliation between the intensity expression of NANOG and MK in OSCC specimens was statistically evaluated using the Chi-squared test, as in our previous report [7]. In addition, the correlation between protein expression patterns and clinicopathological features of OSCC, including tumor stage, neck node metastasis, and histological grade, was also analyzed using the Chi-squared test. The overall survival analysis was determined by using the Kaplan–Meier method, and the data were compared using a log-rank test for the 62 OSCC specimens. First, overall survival analysis was performed according to the treatment modality as well as clinicopathological tumor features, including tumor stage, neck node metastasis, and histological grade (Figure 5A–D).

Second, survival analysis was conducted using the immunostaining intensities of NANOG and MK in the pretreatment biopsy specimens (Figure 6A–C). Univariate survival analyses were conducted using a Kaplan–Meier survival analysis method. In addition, multivariate survival analysis were performed using a Cox proportional hazards regression model. All analyses were performed using IBM SPSS Statistics software (SPSS Inc., Chicago, IL, USA). The cases were censored at the date of either the patient’s death or the last follow-up. Results were considered significant with a *p*-value < 0.05, and any differences were indicated by an asterisk or a different letter.

## 5. Conclusions

In the present study, OSCC patients who have enhanced IHC expression patterns of MK and NANOG in their pretreatment biopsy specimens were significantly associated with lower overall survival rates and unfavorable clinicopathological features. These results demonstrate that analysis of IHC expression patterns of MK and NANOG in pretreatment biopsy specimens during the work-up period can provide a more definitive prognosis prediction for each OSCC patient that can help clinicians to develop a more precise individual treatment modality.

## Figures and Tables

**Table 1:** Summary of clinicopathological features of oral squamous cell carcinoma (OSCCs) and immunohistochemical (IHC) expression patterns of NANOG and MIDKINE.

		NANOG			MIDKINE		
	Total No.	+++	++	+&−	+++	++	+&−
**No. of Cases**	62	21	22	19	37	14	11
**Tumor sites**							
Gingiva	28	11	8	9	17	5	6
Cheek	6	1	3	2	3	2	1
Palate	11	3	6	2	6	5	0
Tongue	10	2	3	5	5	2	3
FOM	7	4	2	1	6	0	1
**Tumor stage**							
I+II	27	6	9	12	14	4	9
III+IV	35	15	13	7	23	10	2
**Neck node**							
Node (−)	39	9	14	16	20	8	11
Node (+)	23	12	8	3	17	6	0
**Histo grade**							
Well	23	4	4	15	8	5	10
Mod + Poor	39	17	18	4	29	9	1

Abbreviations: FOM, floor of mouth; Node (−), negative neck node; Node (+), positive neck node; Well, well-differentiated OSCC; Mod + Poor, moderately- and poorly-differentiated OSCC.

**Table 2:** Univariate (Kaplan–Meier survival analysis) analysis for variable in relation to overall survival of the 62 OSCC patients.

Variable	Overall Survival	
	*p* Value	95% CI
MIDKINE		
MK(+/−)	0.031 *	105.575–150.425
MK(++/+++)		39.938–70.398
NANOG		
NANOG(+/−)	0.022 *	62.674–134.938
NANOG(++/+++)		35.759–68.475
MK + NANOG		
MK(+/−)/NA(+/−)	0.015 *	93.569–137.210
MK(++)/NA(+/−)		34.481–84.387
MK(++)/NA(++)		9.155–20.009
Neck node		
Node (−)	0.001 **	72.644–114.863
Node (+)		14.640–32.900
Histological grade		
Well	0.008 **	75.600–131.275
Moderately		26.664–62.813
Poorly		9.688–48.732
Tumor stage		
Stage I+II	0.023 *	63.238–120.872
Stage III+IV		31.809–67.282
Treatment		
Surg	0.001 **	63.307–115.683
Surg + RT		83.268–137.232
PaRT		9.716–16.344

Abbreviation: CI, confidence interval; Surg, Surgery-only group, Surg + RT, surgery combined with adjuvant radiotherapy or concurrent chemoradiotherapy group, PaRT, palliative radiotherapy or no treatment group; Statistical significance (* *p* < 0.05; ** *p* < 0.01).

**Table 3:** Multivariate Cox proportional hazards regression analysis for variable in relation to overall survival of 62 OSCC patients.

Variable	Overall Survival		
	*p* Value	Hazard Ratio	95% Cl
Neck node	0.004 *	2.091	0.859–5.093
Histological grade	0.003 *	3.570	1.221–10.435
Tumor stage	0.130	2.195	0.057–7.598
NANOG	0.050 *	4.135	2.018–25.037
MIDKINE	0.045 *	3.331	1.079–27.398
MK + NANOG	0.049 *	4.081	2.017–22.266

Abbreviation: Cl, confidence interval; MK + NANOG, Co-expression patterns of MIDKINE and NANOG; Statistical significance (* *p* < 0.05).

## Abbreviations

OSCC	Oral squamous cell carcinoma
MK	Midkine
IHC	Immunohistochemistry or immunohistochemical
P53	Tumor suppress gene p53
OCT4	Octamer-binding transcription factor 4
SOX2	Sex determining region Y-box 2
STAT3	Signal transducer and activator of transcription 3
CSCs	Cancer stem cells
ESCs	Embryonic stem cells
Surg	Surgery-only group
Surg + RT	Surgery combined with adjuvant radiotherapy or concurrent chemoradiotherapy group
PaRT	Palliative radiotherapy or no treatment group
NF-κB	Nuclear factor-κB
DAZL	Deleted in azoospermia-like gene
TTF-1	Thyroid transcription factor-1
VEGF	Vascular endothelial growth factor
